# Soldering with Electricity

**Published:** 1904-11

**Authors:** 


Soldering with Electricity.—The author had recently to repair a denture of gold through which an opposing molar had worn a hole. As one knows, ordinarily this would mean the removing of the teeth, as they were attached to the plate by vulcanite, involving a great deal of work and some elements of risk. I repaired this plate by attaching to it the negative wire of the lighting circuit, and to the positive wire a small carbon, cutting in, in series, a bowl of salt water as a rheostat. The hole in the plate was cleaned and prepared in the usual way, then covered with a piece of foil and 18-k solder placed on it, and borax for the flux. The carbon point was brought in contact with the solder and then gradually removed, forming the arc, which was held sufficiently long to melt the solder. The was closed thereby, and the rubber, hardly an eighth of an inch away, was uninjured; and by immediately immersing the plate in water it did not allow the heat to spread to the surrounding parts. While this case is one that is not common, yet it is well to know that repair of this kind can be made without going through the laborious operation of taking the bite, etc. The melting of any of the metals can be accomplished in the same manner even platinum scrap. I have soldered teeth in this way successfully, and know that by proper care soldering in many cases can be done with electricity.—*Extract, Era.*

